# Health Risks Related to Domestic Violence against Roma Women

**DOI:** 10.3390/ijerph17196992

**Published:** 2020-09-24

**Authors:** Michal Kozubik, Jitse P. van Dijk, Ivan Rac

**Affiliations:** 1 Department of Social Work and Social Sciences, Faculty of Social Sciences and Health Care, Constantine the Philosopher University in Nitra, 949 74 Nitra, Slovakia; 2 Department Community & Occupational Medicine, University of Groningen, University Medical Centre Groningen, 9713 AV Groningen, The Netherlands; j.p.van.dijk@umcg.nl; 3 Graduate School Kosice Institute for Society and Health, Faculty of Medicine, Safarik University, 040 01 Kosice, Slovakia; 4 Olomouc University Social Health Institute, Theological Faculty, Palacky University Olomouc, 771 11 Olomouc, Czech Republic; 5 Institute of Romani Studies, Faculty of Social Sciences and Health Care, Constantine the Philosopher University in Nitra, 949 74 Nitra, Slovakia; irac@ukf.sk

**Keywords:** Roma women, intimate partner violence, handicaps, health consequences

## Abstract

*Background*: Data on Roma women’s experience of violence from their male partners are very scarce. We explored the process of actual domestic violence against Roma women, the threat of violence and its health consequences. We further focused on barriers in the availability of specialized support services aimed at eliminating domestic violence. *Methods*: The sample included 20 Roma women living throughout Slovakia: scattered among the majority (45.0%), in crisis centers and sheltered houses (40.0%), and in segregated Roma settlements (15.0%). Data were obtained through qualitative research by means of semi-structured interviews in 20 individual case studies. All 20 women had experienced a combination of violence: physical, psychological and economic, all of them connected with social isolation. *Results*: Prevailing gender stereotypes are a precondition of domestic violence against women, regardless of their status. Violence against Roma women resulted in several health consequences, and all of the 20 women suffered from these. Most of them reported general psychological problems (75%), among which anxiety and depression (25%), headache (25%), weight loss (10%) and health problems connected with motor activity (5%). The barriers include lack of awareness among Roma women of any specialised support services and the absence of such services for abused women in the region. *Conclusions*: Domestic violence results in serious psychological and physical health consequences. Violence elimination is generally set up without a specific ethnic or gender approach. Disregard of these specifics can lead to deepening of the uneven position of Roma women within the family, community and society, and the acceptance of violence against Roma women.

## 1. Background

The issue of women facing domestic violence is unimaginable for many people [1]. If a woman is beaten, abused or mistreated at home, there is no place where she can find the safety that everyone needs. She is under constant physical and mental stress, which often leads to various psychosomatic problems. Buskotte [2] mentions, among others, cardiovascular and digestive problems, headaches, distractibility, rapid mood changes, depression, sleep disorders and eating disorders.

The important risk factors for physical domestic violence were being a Roma woman (OR = 2.97, 95% CI: 1.44–6.12), living with more than four people in the household (OR = 2.29, 95% CI: 1.21–4.36), being unemployed (OR = 2.15, 95% CI: 1.06–4.37), and getting married only due to her family’s decision (OR = 4.60, 95% CI: 1.42–14.80). The findings confirm that patriarchal and traditional values, women’s lack of financial autonomy, and low socioeconomic status are the risk factors for physical domestic violence [3]. In Roma settlements, 34.8% of men and 23.6% of women believe that under certain circumstances men are justified in being violent towards wives, while among non-Roma, it was 5.6 and 4.0%, respectively. These negative attitudes are significantly associated with lower educational level, lower socio-economic status and being married. Violence prevention activities have to focus on promoting gender equality among youth in vulnerable population groups such as Roma, especially through social support, strengthening their education and employment [4]. Efforts to address this challenge should take an integrated approach, reinforcing the primary health care response to IPV in general, while also promoting more specific actions to address barriers to access affecting all Roma women, and those who experience IPV in particular [5].

Cvikova [6] suggests important steps to be carried out by healthcare professionals to eliminate the violence that women face. Doctors and nurses are often the first professionals visited by an abused woman whose health is affected. If healthcare professionals suspect a woman has been a victim of violence, it is important to enable her to talk without her partner being in close contact with her. For women who have experienced sexual abuse, it is particularly important to be able to consult a female doctor. It is important for patients to know that their interview is absolutely confidential. It is essential to record a woman’s wounds, and to report her state of mind as well. Documentation must be factual. Repeated treatment of injuries, as well as the first treatment of some harm that cannot be labelled as an injury, should warn about the possibility of domestic violence.

A doctor should contact a specialized facility, providing assistance to abused women [7]. In the medical report, the cause of violence is often stated as a “quarrel”. This hides the reality that the man was the perpetrator, and the woman is given the idea that she is actively responsible for the violence. It is important to explore further the cause of the injuries [8]. Health interventions focused on assessing and reducing hostile attributions to children’s behaviours could prevent maternal child abuse in the postnatal period among abused pregnant women [9]. Findings contribute to a growing international body of research, showing the significant effects of interpersonal violence on health and well-being [10].

Roma women in Slovakia live in very differentiated environments; many of them subsist in strong patriarchal structures [11]. They evaluate their environment as a community based on principles supporting the authority of men in relation to women, with the family model of a man as the sole breadwinner and with a limited share of housework and duties [12]. Roma women are discriminated against on the labour market [13]. They have limited or no access to information, the labour market, education and culture [14,15].

The aim of our qualitative study was to explore the process of actual domestic violence against Roma women, the threat of such violence and its health consequences. We further focused on barriers in the availability of specialized support services aimed at eliminating domestic violence.

## 2. Methods

No studies on violence against Roma women in the conditions of the Slovak Republic have been published before. That was the reason why we decided to meet women experiencing violence in person. The qualitative study design allowed us to record their statements in the original form. In contrast to quantitative data collection, we think that the advantages are the in-person contact and verbatim transcripts on the plight of abused women. We used a semi-structured approach [16,17,18,19,20]. Our questions were formulated in such a way that they were not enacting, normative or prescriptive. We planned an adequate time frame for conducting research and field data collection and ensured the confidentiality and anonymity of the participants.

We used a qualitative research design and a semi-structured interview in 20 case studies of Roma women. Our key informant on issues such as age was the head of the Savore non-governmental organization, a Roma woman. This NGO is the most active institution dealing with gender studies and violence against Roma women in Slovakia. We met each of the 20 women in a neutral zone, outside of the household. We tried to separate them from their partners. The interviews were carried out in crisis centres and sheltered houses, and also in the Savore NGO office and in other neutral, informal zones (pubs and restaurants). In order to eliminate the language barrier between the research team and the informants, the interviews were conducted in the Romani language. Thus, the women could express their feelings in their mother tongue. We believe that this fact allowed for a better description of the women’s reality, which also provided higher validity of the collected data.

First of all, we mapped the situation in the sheltered houses and crisis centres which are scattered across the whole country: Western, Eastern and Central Slovakia. All Roma women who explicitly declared their own ethnicity were included in the research. This part of the sample made up 40% (8 women). Then we interviewed the clients of the Savore NGO centre (the activities of the centre are focused on Roma women who face problems with unemployment, social isolation, upbringing and interpersonal violence), and via the snowball method, we encountered other Roma women who had faced domestic violence (approximately 40.0% of clients had met with domestic violence).

### 2.1. Research Sample

Each informant considered herself Roma. The sample included 20 Roma women living in various parts of Slovakia (Table 1).

The study itself was approved by the Ethics Committee of the Ministry for Education, Science, Research and Sport of the Slovak Republic in the framework of VEGA Project no. 1/0111/15; we did not ask the women for written informed consent, but took their participation as an indication of informed consent. Recorded informed consent was obtained from all participants, all of whom agreed to participate in the study, and nobody refused to participate.

### 2.2. Measures

We prepared a detailed anamnesis before the field research and interviews were done. The block of questions consisted of five main parts: demographic information about the woman; information about the offender; relationship issues and the violence process; formal and informal help (e.g., emergency management, social services); conclusions.

Primarily, we mapped domestic violence, specifically intimate partner violence, i.e., violence committed by a man as partner, husband or boyfriend. Secondly, we focused on an abuser, who could be anyone from the close family: father, stepfather, father-in-law, mother or mother-in-law. Finally, we focused on the case that the initiator was a group or community. We particularly focused on those participants who had faced violence and were willing to talk with the researchers about their traumatic experience.

We studied domestic violence, with a focus on its five basic forms: psychological (e.g., lies, criticising, jealousy, self-mutilation, name-calling), economic (e.g., maltreatment, limiting access to belongings, destroying things), sexual (e.g., sexual harassment, rape), physical (e.g., pushing, slapping, kicking, hitting), and social (e.g., isolation, infringement of personal rights, locking in).

### 2.3. Reporting

**Figure 1:**
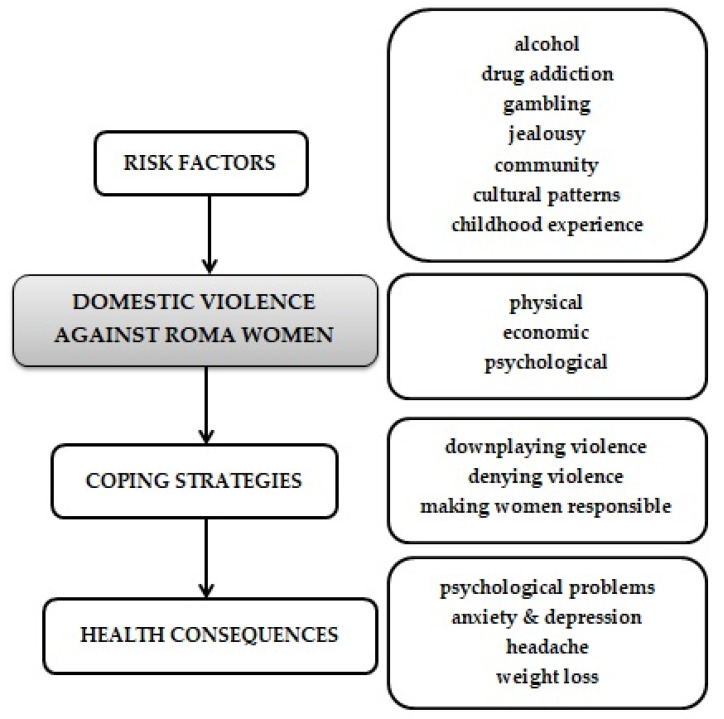
Violence against Roma women in Slovakia.

We used transcripts of the informants’ statements and simple coding to analyse the data. The verbatim statements show the difficult situations that Roma women face. Translations of all quotations were done by the authors. We tried to ensure word-for-word evidence from the Roma women as much as possible. We used transcripts of the informants’ verbatim statements and simple coding to analyse the data. The evaluation of the data was conducted through simple, open coding, followed by the development of central categories (Figure 1). We report the most important statements in verbatim transcriptions of the recordings; they were then translated from Romani and Slovak language into English by the authors of the study.

## 3. Results

### 3.1. Stages: Honeymoon and Structural Violence

At the beginning of each violent relationship is the so-called honeymoon [21] (Box 1). This is a phase of violence when the perpetrator does not behave violently or aggressively; he can control his behaviour to some extent. Everything was “all right” according to the women. Then, when they got married or became partners, the relationship suddenly changed. To some extent, the essence of this whole changing process is the fact that they moved away, were on their own, a child was born, they lived in the mother-in-law’s accommodation, and the partner was sure about his dominance and had a woman in “servitude”. Thus, he could finish the whole process of dominating power over his wife/partner, against whom he started to practise “unbelievable” forms of violence [22].

Box 1. **Stages.**

“…so when he came to our place, at the beginning he behaved well, because I lived with my mom; he only visited me, but when he took me to live in the Czech Republic, he behaved badly to me, he abused me…” (30 years old, 2015).

### 3.2. Risk Factors

The identified triggers of violence include a male partner’s drug addiction. The participants often interpret the possible “cause” of their partners’ violent behaviour as their substance or non-substance abuse, mostly their addiction to playing slot-machines. This addiction cannot be a cause of violence; it is its trigger [23]. This trigger is directly related to economic violence. Jealousy is also a significant impulse, triggering violent behaviour in the partner [24] (Box 2).

Box 2. **Risk factors.**

“…as for alcohol, he often drank, simply every day. Then when he came home drunk, he beat his mother in front of my eyes and as the siblings cried and me being the oldest one, I had to protect both mother and siblings, so you know what it is like” (38 years old, 2015).

“Yes, he was jealous; he made things up about everyone I was in contact with. He swore at me, was vulgar; now I am ashamed to talk about it. He told me bad things; he was jealous of my father too, and so on” (30 years, 2015).

### 3.3. Types of Violence

Verbal attacks are also initiated by the partner, often intentionally. It is one of their strategies to rationalize their violent behaviour. Many of them are annoyed by absolutely minor things. From verbal violence, attacks proceed “smoothly” to psychological violence: extortion, mockery, manipulation and control [25]. After quarrels, jealousy attacks, provocations invoking corresponding reactions from women, there are disproportionate reactions by men, enacted with various forms of violence. The most common are economic, psychological and social violence [26]. They occur in combination, only minimally on their own. We identified manifestations of psychological behaviour, physical violence (including sexual violence) and economic violence (Box 3).

Box 3. **Types of violence.**

Psychological/verbal: “…we must be quiet, as he wants it; peace and quiet… they are often trifles which are common but he is annoyed by them…” (31 years old, 2015).

Psychological: “…he checked if the clothes were sorted by colour, they had to be put in “stacks”, he checked how the bed was made, the sheet had to be stretched—when he threw a coin on it, it had to bounce…” (27 years old, 2015).

Economic: “…he took my gold, took my maternity benefits regularly, simply fraud; he took my ID, then the letters were delivered to me that…then, there was fraud, cameras were taken, I do not know what, it is years, so and such things” (47 years old, 2015).

“…all the money, when I got my maternity benefit, he put it in his pocket and I could not buy anything, anything at all. He said he would come in a while and he did not take me with him; he locked me at home and he went away and came back without money. So many times I begged my family to give me nappies for the little one” (30 years old, 2015).

Physical: “He watched me every minute; when I went to the shops and if I was late there was a quarrel, he wanted to know where I was, who I was with…although there were many people in the queue in front of me…I cannot remember everything now…I could not wear short skirts and dresses at all…everything only below the knees, below the ankles…I could not have a deep neckline, I could not use make-up, not even a bit redder lipstick…only light-coloured lipsticks; even clothes—no vivid colours…” (52 years old, 2015).

Social: “…when I tell him that I am going to the social authorities or that I am going somewhere…he is not happy about it, he asks if I have to go there…and if I have to, he says I have to hurry back…when he comes home from work, he tests me, asks me questions, that he saw me somewhere but in fact he did not see me…or that his friend saw me somewhere…and it is not true…simply there are questions, he asks them as if to catch me…it gets on my nerves…these are really bad moments that I experience when he does such things…he checks the internet…he checks on me in the shop…I can go to the shops only with him…there are many things…” (31 years old, 2015).

Physical: “…he grabbed me with his hands, wrung my mouth with his hands…he grabbed my hair, pulled my hair…” (52 years old, 2015).

Or: “So many times I slept outside, because I was scared to go in because he might almost kill me. He beat me in front of the little one; I had the little one in my arms and he beat me, punched me” (27 years old, 2015).

### 3.4. Relationship-Oriented Violence

One manifestation of violence that the participants face is social violence by isolation [27], a restriction of contacts and relationships with the family and other neighbours. In the case of Roma women, physical violence is not considered to be an undesired phenomenon. Multiple statements by the women reflected this fact, as follows: “If a man does not beat a woman, he does not love her”. In our study, however, we found out that other forms of violence—economic and psychological—are spread much more widely [28].

### 3.5. Children

Almost all of our participants have children with their partners, and even these children do not escape the violence (Box 4). They are not just passive witnesses of violence when they have to see their father’s violent behaviour towards their mother, but they are direct actors in response to their father’s violence. They protect their mothers during physical attacks by their fathers, and thus they are involved directly in the “process of violence”, or they are attacked by their fathers in the same way as their mothers [29].

Box 4. **Children.**

“Yes, me too, the boy too, he grabbed him, raised him up and threw him on the bed, but mostly it was me. And he was a little boy and he shouted at him that he wets himself; he was very annoyed by that” (34 years old, 2015).

### 3.6. Coping with Violence: Downplaying Violence

The participants deal with partner violence in various ways. The most common strategies identified include downplaying the violence, denying the violence and making women responsible for the violence. Downplaying the violence can mean that, after a violent incident, the perpetrator downplays the violence; if he is under the influence of alcohol or drugs, he has a tendency “not to remember anything or what happened”. Violence is also downplayed by the women themselves, which can relate to prevailing gender stereotypes and myths, of which there are many on this issue [30]. Or, on the other hand, they downplay the partner’s violence because of fear of “what could happen”. Violence, however, is also downplayed by the family, acquaintances and neighbours. It is necessary to mention the influence of the mother-in-law on possibly dealing with the situation after a violent incident (Box 5).

Box 5. **Downplaying violence.**

By male: “…I was in shock; I did not know what to do. When he hit me for the first time I wanted to go home immediately. He told me not to go anywhere and to calm down; that nothing had happened and that it was my fault because it was me who had provoked him. So I did not go anywhere, I did not talk to him for about a month” (39 years old, 2015).

By female: “…it cannot happen, no way; the family does not interfere in such things. Because I am to take care of children and he is to get drunk and beat his wife” (30 years old, 2015).

By mother-in-law: “It is absolutely normal, get used to it; I had to get used to that, too” (Stojka, Pivoň 2003) and “I just screamed, we lived at his mother’s and his mother was on his side, and she saw he was beating me, this, and she encouraged him…” (27 years old, 2015).

By family: “…and as for the family, deaf and blind…I cannot say anything else about it…they try to see nothing…it is always easier when I do not see, feel…” (31 years old, 2015).

### 3.7. Coping with Violence: Denying the Violence

Besides the downplaying of violence, we also found the issue of “denying” the violence, when the partner absolutely denies that there have been any incidents (Box 6). Women start to believe what their partners say, and based on their power, they accept the principle that violence against them is something normal. One of the important moments in denying and downplaying the violence of the partner is the perception of the public and the neighbourhood. The paradox [31] is that “outwardly”, the man is very nice, the one who helps everyone, the one who would never hurt anybody, a model father and husband. Such a partner is called a manipulator, one who can get other people to take “his side”.

Box 6. **Denying violence.**

“His family and people in the neighbourhood considered him a very gentle, sensitive husband who was always able to take care of his family, went for walks with the children, was a role model and what was happening at home, I will tell you the truth, it was like in a pot under a lid. Only those who took the lid off could see what was in the pot. But nobody came to take that lid off. So, at the time, he was the very best man” (56 years old, 2015).

### 3.8. Coping with Violence: Making Women Responsible for Violence

This manifestation of a partner’s power and domination over the woman means that, despite many “attempts” to leave the partner, the women stay in the relationships. The most common reasons are economic dependence and fear, the belief that the partner could change, and the absence of help and support to get out of the relationship (Box 7). A very common way of “resolving” violence by the family is ignoring it or making it a public secret [32]. They have a tendency to hurt any woman who brings her experience with violence “to light”; thus, she is exposed to secondary victimisation. We found the phenomenon of unawareness and fear to be a barrier to seeking help. Only a few women were able to talk publicly about their experience.

Box 7. **Making women responsible for violence.**

Change: “…I hoped that he would change, but no change came; it also damaged my marriage, and so I went down the drain because I was stupid that I…blind love and then I found out that this partner was not for me…but even if I forgave him all the time, all that infidelity, all the beating up, but I was not sure about him and I was desperate because of that relationship, I wanted to change him, but he never listened to me…” (38 years old, 2015).

Victimisation: “And it really is difficult for me, because when I had nobody to talk to about it and I had to be quiet and suffer his sick sexual desires. Gradually, his mother started to notice something—that he often came to me but she did not pay any attention that something was going on between him and me, even though she suspected something, but she only let it go” (38 years old, 2015).

Barrier: “…nobody was able to give me advice, or to direct me, I did not know there was something where they could help me… I had a lack of information about all this” (52 years old, 2015).

### 3.9. Consequences of Violence

The last, but not least, issue identified concerns the consequences of the violence experienced by the women and their children. Health consequences are mentioned in almost every transcript, and they relate closely to psychological effects, both on women and their children (Box 8). Violence against Roma women results in several health consequences, and all of the 20 women suffered from these. Most of them reported general psychological problems (75%), including anxiety and depression (25%), headache (25%), weight loss (10%) and health problems connected with motor activity (5%).

Box 8. **Consequences of violence.**

“My health status…I feel, feel very exhausted after all that I went through, especially my psyche, and I have problems with the daughter, too. As for her behaviour, she dealt with this very badly…I became very mistrustful of men, and also I do not believe other people a lot and I am scared to let people get into my heart. And I do not want to have a relationship now. I need to put myself back together”. (38 years old, 2015).

“I started visiting a psychologist, and I go there, but now I do not need it; I am all right, well all right like all right; here inside me it will still be there as long as I live, but I have already…slowly I am getting over it, I try, I am short-tempered. I have become not aggressive but short-tempered; I was stoic, but I have become what I am now just because of him” (47 years old, 2015).

“Yes, I have, as for this…and I have some prescribed medicaments that I have to take; they are antidepressants so that I can manage at least a little with the children” (34 years old, 2015).

“…the last quarrel finished with him calling an ambulance and saying that I was aggressive. When they arrived I was scared, because police came too; they took me out with the handcuffs on because I was crying for help… My family did not know about anything. I was at the psychiatric department for three weeks; they gave me sleeping pills” (27 years, 2015).

We consider it very important that a functioning network of social services should be established all over the country, and that this provision will not be restricted to the bigger cities or towns. Nowadays, women living in segregated settlements have no opportunity to get help for their problems in interpersonal domestic violence.

## 4. Discussion

We focused on barriers in the availability of specialized support services aimed at eliminating domestic violence. Roma women have a lack of information on specialized services and faced poor access to them.

We found that the very start a relationship was not always violent, and that the risk factors were alcohol or other forms of addiction. As types of violence, we identified verbal, psychological, physical, including sexual violence, and economic violence. Children played a role in the violent relationship. The most common identified strategies included downplaying the violence, denying the violence and making women responsible for the violence. Finally, we found several physical and mental health consequences.

We identified the phases of violence [33,34,35] through all the triggers that “start” the process of violence; substance and non-substance addictions, jealousy and gender stereotypes, which are not the causes of violence but its catalysts and, unfortunately, excusing and legitimising violence. We furthermore found the manifestations of economic and social violence related to excessive control.

Although our sample cannot be considered statistically representative, we found first of all a combination of economic and social violence related to excessive control. The interpretation of our finding is similar to that by Bodnárová and Filadelfiová [1]; we suggest terming this a paradox of committed violence. The environmental conditions, i.e., low standards of housing and poor access to services, high rates of gender stereotypes, prejudices and a rather high threshold of sensitivity to violence, led to the manifestations “typical” for the environment of the middle and higher working social classes. The perpetrator has at least secondary education, is employed and commits violence in more sophisticated ways, so that the “signs of violence” are as minimally visible as possible [36].

We found that downplaying violence is one of the more frequent strategies for dealing with “violent situations” that women use, and at a much higher rate, their partners themselves as perpetrators. The women seek rational reasons in their downplaying; the men use it as a well-prepared strategy to hide violence from people “outside”. In all cases, the family and neighbourhood are deliberate “supernumeraries” who rather seek a reason not to help in the situation than a way to help the woman and children get out of the relationship [37]. The downplaying of violence is a common part of the cycle of violence [21].

The women stay in their relationships despite the violence due to pressure from the neighbourhood and family, the lack of help and the influence of the perpetrators. Cirtkova, Vitousova et al. [38] explain this as Stockholm Syndrome [39]. Outsiders are led to believe that the staying women “encourage” the partners into thinking that they “can commit violence” because nobody stops them. In many cases, not even the police called to the “crime” scene, or the experts who want to help, actually stop him; in spite of all of this, the women decide to persist in living with the perpetrator. The reasons for staying might be fear, extortion, threats that he may hurt their children and/or family, worries about the future, and pressure from the community. In general, the more marginalized the community, the smaller the chance of escaping the vicious circle [40]. The consequences of this process include physical and mental health problems in women facing violence and in their children.

It is not possible to talk about equal positions of men and women in the subethnic group of Vlachiko Roma, otherwise the fundamental principles of functioning of their community would be violated. The expected ideal is a woman who is able to take care of the children, husband and household, and lives her life in accordance with the Roma principles, which among other things, means that she is obedient and loyal to her husband, and thus she shows him respect [11].

### 4.1. Strengths and Limitations

This study is unique in that no similar research focusing on intimate partner violence in Roma women in marginalised communities has been conducted in Slovakia. Some limitations must be considered as well, however. Our sample consisted of 20 women, some of them living in a facility, some of them not. Regardless of this, the male partner was never present during the interviews, otherwise the participants might not have felt free to talk about their past; moreover, the interviews were carried out in the Romany language. Furthermore, the idiographic approach in social sciences was used.

### 4.2. Implications

Field social workers should improve their knowledge with regard to domestic violence within education programmes provided by experts from non-governmental organizations, human rights organisations or universities, for example. The multi-institutional cooperation of field social workers with government, public administration, local authorities and law enforcement agencies is indispensable. Since Roma women living in marginalized communities are socially excluded, it is necessary to provide them with free and safe access to information related to violence, its prevention and the services available. We recommend creating an accessible network of social services, so that each victim of violence is provided with counselling, assistance and/or emergency housing.

We also recommend studying Roma women from the Vlachiko community, because the character of this work might significantly differ from the findings in the Rumungro community [41,42,43]. A woman’s status in a partnership is strictly defined by the culture: the man’s status is always dominant, and the woman cannot talk to other men, for example. We therefore recommend including female researchers with basic knowledge of the Vlachiko dialect of the Roma language [11].

Future research should explore whether domestic violence follows a similar pattern, as we report here in smaller units like villages.

## 5. Conclusions

We found that the course and the impacts of violence on the minority of Roma women do not differ from the progress, strategies, manifestation and sorts of acts of violence committed on women in the majority [35,44]. Future research should focus on exploring whether domestic violence follows a similar pattern, as we report here in smaller units like villages. We also found that the statement: “the poorer, the more physical violence” [45] is not valid any more for Roma women in Slovakia. Violence occurs in any social class, affecting all women, regardless of age, religion, social status, education or nationality/ethnicity [1]. However, the situation is different for Rumungro Roma women living in communities in Eastern Slovakia, who face quadruple marginalization, i.e., living in the area with the highest unemployment rates, living in segregated settlements, being Roma, and being women who may face domestic violence. The study draws attention to this highly unfavourable situation. The superior position of men to women relates also to gender differences, when taking place in different spaces under different circumstances that the woman experiences in multiple situations throughout her life. Strict endogamy and social control are evident in the women’s lives from childhood in the community of Vlachiko Roma [8].

## Figures and Tables

**Table 1:** Participants by age, marital status and type of living and region.

Regions	Western Slovakia	Central Slovakia	Eastern Slovakia
Women	8 (40.0%)	7 (35.0%)	5 (25.0%)
Age (years)	27–59	30–65	19–45
Marital status	Not available	Married 2 (10.0%)	Married 3 (15.0%)
		Living with partner 5 (25.0%)	Divorced 2 (10.0%)
Type of living	Emergency	Segregated 2 (10.0%)	Segregated 1 (5.0%)
	(40.0%)	Scattered 5 (25.0%)	Scattered 4 (20.0%)
Towns	Bratislava	Banska Bystrica	Kezmarok
	Lozorno	Banska Stiavnica	Poprad
		Brezno	Spisska Nova Ves
		Hronsky Benadik	Stara Lubovna
		Ziar nad Hronom	
